# 5-Hydr­oxy-1-(3-hydr­oxy-2-naphtho­yl)-3,5-dimethyl-2-pyrazoline

**DOI:** 10.1107/S1600536808023027

**Published:** 2008-07-31

**Authors:** Yuting Chen, Da-Cheng Li, Yuehua Zhu, Da-Qi Wang

**Affiliations:** a Department of Chemistry, Dezhou University, Dezhou 253023, People’s Republic of China; b College of Chemistry and Chemical Engineering, Liaocheng University, Liaocheng 252059, People’s Republic of China; c School of Materials Science and Engineering, Liaocheng University, Liaocheng 252059, People’s Republic of China

## Abstract

In the title mol­ecule, C_16_H_16_N_2_O_3_, intra­molecular O—H⋯O hydrogen bonds influence the mol­ecular conformation. Inter­molecular O—H⋯O hydrogen bonds [O⋯O = 2.922 (2) Å] link the mol­ecules into centrosymmetric dimers. Weak inter­molecular C—H⋯O inter­actions assemble these dimers into layers parallel to the *bc* plane.

## Related literature

A highly puckered 60-membered metalladiaza­macrocycle was reported by Moon *et al.* (2006 ▶), and two manganese metallacrowns with the ligand *N*-acyl-3-hydr­oxy-2-naphthalene­carbohydrazide were reported by Dou *et al.* (2006 ▶). The ligand 1-benzoyl-3,5-dimethyl-5-(1-benzoyl­hydrazido)pyrazoline was first synthesized by Mukhopadhyay & Pal (2004 ▶).
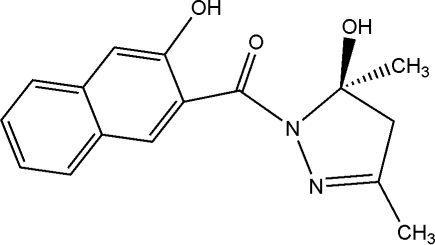


## Experimental

### 

#### Crystal data

C_16_H_16_N_2_O_3_
*M* *_r_* = 284.31
Monoclinic, 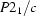

*a* = 12.368 (6) Å
*b* = 7.428 (4) Å
*c* = 17.041 (9) Å
β = 109.331 (7)°
*V* = 1477.3 (13) Å^3^
*Z* = 4
Mo *K*α radiation
μ = 0.09 mm^−1^
*T* = 298 (2) K
0.64 × 0.57 × 0.39 mm

#### Data collection

Bruker SMART 1000 CCD area-detector diffractometer
Absorption correction: multi-scan (*SADABS*; Sheldrick, 1996 ▶) *T*_min_ = 0.945, *T*_max_ = 0.966
7363 measured reflections
2588 independent reflections
1627 reflections with *I* > 2σ(*I*)
*R*_int_ = 0.042

#### Refinement

*R*[*F* ^2^ > 2σ(*F* ^2^)] = 0.044
*wR*(*F* ^2^) = 0.130
*S* = 1.00
2588 reflections
190 parameters
H-atom parameters constrained
Δρ_max_ = 0.15 e Å^−3^
Δρ_min_ = −0.18 e Å^−3^

### 

Data collection: *SMART* (Siemens, 1996 ▶); cell refinement: *SAINT* (Siemens, 1996 ▶); data reduction: *SAINT*; program(s) used to solve structure: *SHELXS97* (Sheldrick, 2008 ▶); program(s) used to refine structure: *SHELXL97* (Sheldrick, 2008 ▶); molecular graphics: *SHELXTL* (Sheldrick, 2008 ▶); software used to prepare material for publication: *SHELXTL*.

## Supplementary Material

Crystal structure: contains datablocks I, global. DOI: 10.1107/S1600536808023027/cv2430sup1.cif

Structure factors: contains datablocks I. DOI: 10.1107/S1600536808023027/cv2430Isup2.hkl

Additional supplementary materials:  crystallographic information; 3D view; checkCIF report

## Figures and Tables

**Table 1:** Hydrogen-bond geometry (Å, °)

*D*—H⋯*A*	*D*—H	H⋯*A*	*D*⋯*A*	*D*—H⋯*A*
O2—H2⋯O1	0.82	1.79	2.518 (2)	147
O3—H3⋯O1	0.82	2.36	2.888 (2)	122
O3—H3⋯O2^i^	0.82	2.27	2.922 (2)	137
C9—H9⋯O3^ii^	0.93	2.57	3.388 (3)	147

Symmetry codes: (i) 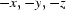; (ii) 
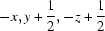
.

**Fig. 1.**
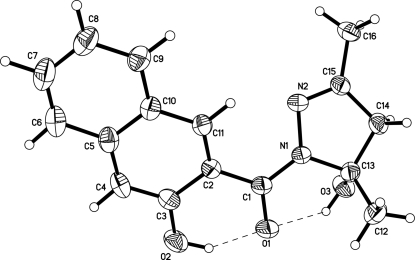
The molecular structure of the title compound showing the atomic numbering and 30% probability displacement ellipsoids. Dashed lines denote hydrogen bonds.

## supplementary crystallographic information

### Comment

Aroylhydrazine ligands have gained an increasing attraction due to their interesting chemical activities (Moon *et al.*, 2006). As an extension of our work on the structural characterization of aroylhydrazine derivatives, along with our work of successful assembly of two azametallacrowns using *N*-acyl-3-hydroxy-2-naphthalenecarbohydrazide (Dou *et al.*, 2006), the title compound, (I), was synthesized and characterized.

Pyrazoline ring in (I) is nearly co-planar with the mean deviation of 0.0379 Å from its least-squares plane, and the distances of N1—N2, C13—N1 and C15—N2 are 1.403 (2), 1.498 (2) and 1.275 (2) Å, respectively, which are in agreement with those of the analogous compound (Mukhopadhyay & Pal, 2004). The dihedral angle between the pyrazoline ring and naphthalene ring is 28.2 (3)°.

There are intramolecular O2—H2···O1 and O3—H3···O1 hydrogen bonds (Table 1, Fig. 1), which influence the molecular conformation. The intermolecular O—H···O (Table 1) hydrogen bonds link molecules into centrosymmetric dimers, and the weak intermolecular C—H···O interactions (Table 1) assemble further these dimers into the layers parallel to *bc*-plane.

### Experimental

0.21 ml of acetylacetone (0.205 g, 2.05 mmol) were added into a methanol solution (15 ml) of 3-hydroxy-2-naphthoylhydrazine (0.404 g, 2 mmol). The mixture was refluxed for 3 h followed by evaporation to approximate 1/3 of the original volume on a rotary evaporator, then the solution was cooled to room temperature. After the solution was allowed to stand for 2 weeks, yellow block crystals suitable for X-ray structure determination was obtained. Yield: 0.400 g, 70.37%. m.p.: 565–567 K. Anal. for C_16_H_16_N_2_O_3_: Calc. C, 67.53; H, 5.63; N, 9.85; Found: C, 67.20; H, 5.49; N, 9.28%. The No. of CCDC: 693975.

### Refinement

All H atoms were placed in geometrically idealized positions (C—H 0.93–0.96 Å, O—H 0.82 Å) and treated as riding on their parent atoms, with U_iso_(H)= 1.2–1.5U_eq_ of the parent atom.

### Crystal data

**Table:**

C_16_H_16_N_2_O_3_	*F*_000_ = 600
*M**_r_* = 284.31	*D*_x_ = 1.278 Mg m^−^^3^
Monoclinic, *P*2_1_/*c*	Mo *K*α radiation λ = 0.71073 Å
*a* = 12.368 (6) Å	Cell parameters from 2043 reflections
*b* = 7.428 (4) Å	θ = 2.5–23.0º
*c* = 17.041 (9) Å	µ = 0.09 mm^−^^1^
β = 109.331 (7)º	*T* = 298 (2) K
*V* = 1477.3 (13) Å^3^	Block, yellow
*Z* = 4	0.64 × 0.57 × 0.39 mm

### Data collection

**Table:**

Bruker SMART 1000 CCD area-detector diffractometer	2588 independent reflections
Radiation source: fine-focus sealed tube	1627 reflections with *I* > 2σ(*I*)
Monochromator: graphite	*R*_int_ = 0.042
*T* = 298(2) K	θ_max_ = 25.0º
φ and ω scans	θ_min_ = 2.6º
Absorption correction: multi-scan(SADABS; Sheldrick, 1996)	*h* = −12→14
*T*_min_ = 0.945, *T*_max_ = 0.966	*k* = −8→8
7363 measured reflections	*l* = −20→19

### Refinement

**Table:**

Refinement on *F*^2^	Secondary atom site location: difference Fourier map
Least-squares matrix: full	Hydrogen site location: inferred from neighbouring sites
*R*[*F*^2^ > 2σ(*F*^2^)] = 0.044	H-atom parameters constrained
*wR*(*F*^2^) = 0.130	*w* = 1/[σ^2^(*F*_o_^2^) + (0.0553*P*)^2^ + 0.3243*P*] where *P* = (*F*_o_^2^ + 2*F*_c_^2^)/3
*S* = 1.00	(Δ/σ)_max_ < 0.001
2588 reflections	Δρ_max_ = 0.15 e Å^−^^3^
190 parameters	Δρ_min_ = −0.18 e Å^−^^3^
Primary atom site location: structure-invariant direct methods	Extinction correction: none

### Special details

**Table:**

Geometry. All e.s.d.'s (except the e.s.d. in the dihedral angle between two l.s. planes) are estimated using the full covariance matrix. The cell e.s.d.'s are taken into account individually in the estimation of e.s.d.'s in distances, angles and torsion angles; correlations between e.s.d.'s in cell parameters are only used when they are defined by crystal symmetry. An approximate (isotropic) treatment of cell e.s.d.'s is used for estimating e.s.d.'s involving l.s. planes.
Refinement. Refinement of *F*^2^ against ALL reflections. The weighted *R*-factor *wR* and goodness of fit *S* are based on *F*^2^, conventional *R*-factors *R* are based on *F*, with *F* set to zero for negative *F*^2^. The threshold expression of *F*^2^ > σ(*F*^2^) is used only for calculating *R*-factors(gt) *etc*. and is not relevant to the choice of reflections for refinement. *R*-factors based on *F*^2^ are statistically about twice as large as those based on *F*, and *R*- factors based on ALL data will be even larger.

### Fractional atomic coordinates and isotropic or equivalent isotropic displacement parameters (Å^2^)

**Table:**

	*x*	*y*	*z*	*U*_iso_*/*U*_eq_	
N1	−0.04413 (13)	0.2580 (2)	0.17142 (9)	0.0490 (5)	
N2	−0.01893 (14)	0.2214 (2)	0.25642 (10)	0.0518 (5)	
O1	−0.00569 (12)	0.2318 (2)	0.05376 (8)	0.0685 (5)	
O2	0.18079 (13)	0.1067 (2)	0.04814 (9)	0.0751 (5)	
H2	0.1117	0.1265	0.0322	0.113*	
O3	−0.21475 (12)	0.1233 (2)	0.08086 (9)	0.0660 (5)	
H3	−0.1868	0.1116	0.0437	0.099*	
C1	0.03222 (17)	0.2371 (3)	0.13121 (12)	0.0492 (5)	
C2	0.15776 (16)	0.2220 (3)	0.17539 (12)	0.0459 (5)	
C3	0.22724 (19)	0.1534 (3)	0.12957 (13)	0.0547 (6)	
C4	0.34226 (19)	0.1307 (3)	0.16761 (15)	0.0650 (6)	
H4	0.3857	0.0837	0.1372	0.078*	
C5	0.39654 (18)	0.1758 (3)	0.25100 (15)	0.0585 (6)	
C6	0.5152 (2)	0.1459 (4)	0.29304 (19)	0.0809 (8)	
H6	0.5599	0.0937	0.2648	0.097*	
C7	0.5633 (2)	0.1923 (4)	0.3733 (2)	0.0918 (9)	
H7	0.6409	0.1707	0.3998	0.110*	
C8	0.4998 (2)	0.2720 (4)	0.41801 (17)	0.0803 (8)	
H8	0.5351	0.3034	0.4735	0.096*	
C9	0.38560 (18)	0.3038 (3)	0.38005 (14)	0.0639 (6)	
H9	0.3434	0.3579	0.4097	0.077*	
C10	0.33104 (16)	0.2550 (3)	0.29614 (13)	0.0510 (5)	
C11	0.21191 (16)	0.2757 (3)	0.25610 (12)	0.0478 (5)	
H11	0.1685	0.3277	0.2854	0.057*	
C12	−0.19627 (19)	0.4480 (3)	0.07493 (14)	0.0704 (7)	
H12A	−0.1643	0.5503	0.1092	0.106*	
H12B	−0.2778	0.4624	0.0510	0.106*	
H12C	−0.1634	0.4385	0.0314	0.106*	
C13	−0.17009 (16)	0.2794 (3)	0.12726 (12)	0.0512 (5)	
C14	−0.21392 (17)	0.2823 (3)	0.20069 (12)	0.0561 (6)	
H14A	−0.2422	0.4008	0.2078	0.067*	
H14B	−0.2750	0.1952	0.1932	0.067*	
C15	−0.11177 (18)	0.2339 (3)	0.27323 (12)	0.0503 (5)	
C16	−0.1148 (2)	0.2027 (4)	0.35844 (13)	0.0700 (7)	
H16A	−0.0392	0.1736	0.3948	0.105*	
H16B	−0.1658	0.1048	0.3576	0.105*	
H16C	−0.1413	0.3096	0.3780	0.105*	

### Atomic displacement parameters (Å^2^)

**Table:**

	*U*^11^	*U*^22^	*U*^33^	*U*^12^	*U*^13^	*U*^23^
N1	0.0456 (9)	0.0637 (12)	0.0350 (9)	0.0036 (8)	0.0099 (7)	−0.0026 (8)
N2	0.0550 (10)	0.0632 (12)	0.0363 (9)	0.0032 (9)	0.0139 (8)	−0.0016 (8)
O1	0.0661 (10)	0.1004 (14)	0.0375 (9)	0.0037 (9)	0.0149 (7)	−0.0036 (8)
O2	0.0795 (11)	0.0951 (13)	0.0596 (10)	−0.0029 (10)	0.0349 (8)	−0.0186 (9)
O3	0.0613 (9)	0.0802 (12)	0.0583 (9)	−0.0128 (8)	0.0220 (7)	−0.0254 (8)
C1	0.0543 (12)	0.0527 (13)	0.0402 (12)	−0.0009 (10)	0.0149 (10)	−0.0026 (10)
C2	0.0477 (11)	0.0455 (12)	0.0458 (12)	−0.0003 (9)	0.0172 (9)	0.0000 (9)
C3	0.0618 (14)	0.0525 (14)	0.0552 (14)	−0.0047 (11)	0.0264 (11)	−0.0050 (11)
C4	0.0583 (14)	0.0659 (16)	0.0843 (18)	0.0027 (12)	0.0418 (13)	−0.0087 (13)
C5	0.0487 (12)	0.0512 (14)	0.0780 (16)	−0.0018 (10)	0.0241 (11)	0.0009 (12)
C6	0.0516 (15)	0.083 (2)	0.111 (2)	0.0078 (13)	0.0310 (15)	−0.0011 (17)
C7	0.0461 (14)	0.110 (2)	0.109 (2)	0.0047 (15)	0.0115 (16)	0.006 (2)
C8	0.0534 (15)	0.092 (2)	0.0789 (18)	−0.0047 (14)	0.0001 (13)	0.0033 (15)
C9	0.0519 (13)	0.0683 (16)	0.0643 (15)	−0.0033 (11)	0.0095 (11)	−0.0032 (12)
C10	0.0463 (12)	0.0437 (13)	0.0617 (14)	−0.0021 (9)	0.0159 (10)	0.0017 (10)
C11	0.0467 (11)	0.0460 (12)	0.0518 (12)	0.0014 (9)	0.0177 (9)	−0.0011 (10)
C12	0.0645 (14)	0.0791 (18)	0.0578 (14)	0.0139 (13)	0.0072 (11)	0.0067 (13)
C13	0.0442 (11)	0.0611 (14)	0.0422 (12)	0.0014 (10)	0.0059 (9)	−0.0105 (10)
C14	0.0513 (12)	0.0642 (15)	0.0525 (13)	0.0044 (10)	0.0169 (10)	−0.0115 (11)
C15	0.0556 (13)	0.0515 (13)	0.0450 (12)	0.0024 (10)	0.0184 (10)	−0.0088 (10)
C16	0.0845 (17)	0.0810 (18)	0.0526 (14)	0.0132 (14)	0.0337 (12)	−0.0003 (12)

### Geometric parameters (Å, °)

**Table:**

N1—C1	1.347 (3)	C7—C8	1.393 (4)
N1—N2	1.404 (2)	C7—H7	0.9300
N1—C13	1.497 (3)	C8—C9	1.366 (3)
N2—C15	1.276 (3)	C8—H8	0.9300
O1—C1	1.246 (2)	C9—C10	1.412 (3)
O2—C3	1.360 (2)	C9—H9	0.9300
O2—H2	0.8200	C10—C11	1.412 (3)
O3—C13	1.409 (2)	C11—H11	0.9300
O3—H3	0.8200	C12—C13	1.509 (3)
C1—O1	1.246 (2)	C12—H12A	0.9600
C1—C2	1.489 (3)	C12—H12B	0.9600
C2—C11	1.375 (3)	C12—H12C	0.9600
C2—C3	1.432 (3)	C13—C14	1.520 (3)
C3—C4	1.365 (3)	C14—C15	1.490 (3)
C4—C5	1.397 (3)	C14—H14A	0.9700
C4—H4	0.9300	C14—H14B	0.9700
C5—C10	1.416 (3)	C15—C16	1.483 (3)
C5—C6	1.421 (3)	C16—H16A	0.9600
C6—C7	1.344 (4)	C16—H16B	0.9600
C6—H6	0.9300	C16—H16C	0.9600
			
C1—N1—N2	123.32 (16)	C9—C10—C11	122.3 (2)
C1—N1—C13	122.99 (16)	C9—C10—C5	119.29 (19)
N2—N1—C13	112.21 (15)	C11—C10—C5	118.4 (2)
C15—N2—N1	107.99 (16)	C2—C11—C10	122.45 (19)
C3—O2—H2	109.5	C2—C11—H11	118.8
C13—O3—H3	109.5	C10—C11—H11	118.8
O1—C1—N1	117.46 (18)	C13—C12—H12A	109.5
O1—C1—C2	119.79 (18)	C13—C12—H12B	109.5
N1—C1—C2	122.75 (17)	H12A—C12—H12B	109.5
C11—C2—C3	117.85 (18)	C13—C12—H12C	109.5
C11—C2—C1	124.41 (18)	H12A—C12—H12C	109.5
C3—C2—C1	117.69 (18)	H12B—C12—H12C	109.5
O2—C3—C4	118.35 (19)	O3—C13—N1	110.11 (16)
O2—C3—C2	121.35 (19)	O3—C13—C12	112.56 (17)
C4—C3—C2	120.3 (2)	N1—C13—C12	111.68 (18)
C3—C4—C5	121.9 (2)	O3—C13—C14	107.04 (18)
C3—C4—H4	119.1	N1—C13—C14	100.54 (15)
C5—C4—H4	119.1	C12—C13—C14	114.20 (19)
C4—C5—C10	118.92 (19)	C15—C14—C13	104.14 (17)
C4—C5—C6	122.9 (2)	C15—C14—H14A	110.9
C10—C5—C6	118.1 (2)	C13—C14—H14A	110.9
C7—C6—C5	120.6 (2)	C15—C14—H14B	110.9
C7—C6—H6	119.7	C13—C14—H14B	110.9
C5—C6—H6	119.7	H14A—C14—H14B	108.9
C6—C7—C8	121.7 (2)	N2—C15—C16	121.66 (19)
C6—C7—H7	119.1	N2—C15—C14	114.22 (18)
C8—C7—H7	119.1	C16—C15—C14	124.1 (2)
C9—C8—C7	119.7 (3)	C15—C16—H16A	109.5
C9—C8—H8	120.1	C15—C16—H16B	109.5
C7—C8—H8	120.1	H16A—C16—H16B	109.5
C8—C9—C10	120.5 (2)	C15—C16—H16C	109.5
C8—C9—H9	119.7	H16A—C16—H16C	109.5
C10—C9—H9	119.7	H16B—C16—H16C	109.5

### Hydrogen-bond geometry (Å, °)

**Table:**

*D*—H···*A*	*D*—H	H···*A*	*D*···*A*	*D*—H···*A*
O2—H2···O1	0.82	1.79	2.518 (2)	147
O3—H3···O1	0.82	2.36	2.888 (2)	122
O3—H3···O2^i^	0.82	2.27	2.922 (2)	137
C9—H9···O3^ii^	0.93	2.57	3.388 (3)	147

Symmetry codes:  (i) −*x*, −*y*, −*z*; (ii) −*x*, *y*+1/2, −*z*+1/2.
